# Comprehensive Analysis of *Sterol O-Acyltransferase 1* as a Prognostic Biomarker and Its Association With Immune Infiltration in Glioma

**DOI:** 10.3389/fonc.2022.896433

**Published:** 2022-05-12

**Authors:** Xuyang Guo, Shaolong Zhou, Zhuo Yang, Zi-An Li, Weihua Hu, Lirui Dai, Wulong Liang, Xinjun Wang

**Affiliations:** ^1^ Department of Neurosurgery, The Fifth Affiliated Hospital of Zhengzhou University, Zhengzhou, China; ^2^ Henan International Joint Laboratory of Glioma Metabolism and Microenvironment Research, Zhengzhou, China

**Keywords:** SOAT1, glioma, immune infiltration, prognosis, cholesterol metabolism

## Abstract

Metabolic reprogramming is a hallmark of glioma, and *sterol O-acyltransferase 1 (SOAT1)* is an essential target for metabolic therapy. However, the prognostic value of *SOAT1* and its association with immune infiltration has not been fully elucidated. Using RNA-seq and clinical data of glioma patients from The Cancer Genome Atlas (TCGA), *SOAT1* was found to be correlated with poor prognosis in glioma and the advanced malignancy of clinicopathological characteristics. Next, the correlation between *SOAT1* expression and tumor-infiltrating immune cells was performed using the single-sample GSEA algorithm, gene expression profiling interactive analysis (GEPIA), and tumor immune estimation resource version 2 (TIMER2.0); it was found that *SOAT1* expression was positively correlated with multiple tumor-infiltrating immune cells. To further verify these results, immunofluorescence was conducted on paraffin-embedded glioma specimens, and a positive trend of the correlation between *SOAT1* expression and Treg infiltration was observed in this cohort. Finally, differentially expressed gene analysis, and Gene Ontology and Kyoto Encyclopedia of Genes and Genomes analyses were performed to explore the biological processes and signaling pathways that *SOAT1* may be involved in during glioma pathogenesis. A protein-protein interaction network was established, and co-expression analysis was conducted to investigate the regulatory mechanism of *SOAT1* in glioma. To the best of our knowledge, this is the first comprehensive study reporting that *SOAT1* may serve as a novel prognostic biomarker associated with immune infiltrates, providing a novel perspective for glioma metabolic therapy.

## Introduction

Glioma is the most common type of primary tumor in the adult central nervous system, and glioblastoma (GBM) is the most commonly occurring malignant brain tumor. Despite receiving standard therapeutic regimens, including surgical resection, targeted radiation therapy, and chemotherapy, the prognosis of glioma remains unsatisfactory (1). Immunotherapy has emerged as a novel strategy for cancer treatment and has been used successfully for various cancer types, creating new opportunities for glioma treatment (2). However, clinical trials on immunotherapy have thus far failed to report encouraging results (3–6). The unique immune environment in the brain and tumor microenvironment (TME) comprise the main barrier of immunotherapy (7). Furthermore, metabolic reprogramming is a hallmark of glioma. Metabolic remodeling has been found to markedly impact the TME (8), which promotes tumor progression and immunosuppression (9). Therefore, metabolic therapy is a promising approach for ameliorating the TME and improving the efficacy of immunotherapy.

The *sterol O-acyltransferase 1* (*SOAT1*) gene was mapped to the human chromosome 1q25. *SOAT1* protein contains nine transmembrane domains (TMD) and the catalytic His460 residue is involved in free cholesterol binding in TMD7, with a portion of the N-terminal domain and TMD8 being important for subunit interactions (10). *SOAT1* is a key enzyme that acts to esterify the 3-hydroxyl position of cellular free cholesterol with a fatty acid-CoA creating cholesteryl ester (CE) (11). CE is stored as lipid droplets (LDs) in cells. Tumor cells exhibit metabolic abnormalities to meet the elevated energy and biosynthetic demands associated with rapid tumor growth (12), and thus *SOAT1* has become an essential target of metabolic therapy for tumor treatment. Thus far, *SOAT1* has exhibited a prognostic value in several types of cancers, and its blockade can inhibit the proliferation of neoplasm cells in various cancer types, including pancreatic cancer, glioma, prostate cancer, lung cancer, and adrenocortical carcinoma (13–17).

Lipid metabolism reprogramming promotes immune suppression by facilitating immune infiltration (9). LD accumulation and increased *SOAT1* expression have been discovered in glioma (14), whereas the relationship between *SOAT1* expression and immune infiltration in glioma remains unclear. The aim of the present study was to explore the association between *SOAT1* expression and immune infiltration using bioinformatics analysis. In addition, since T regulatory cells (Treg) are important cells that exert an anti-immune effect in TME (8), the correlation between *SOAT1* expression and Treg infiltration were further explored using immunofluorescence. The prognostic value of *SOAT1* in glioma has not been fully elucidated, so it was explored herein. In addition, the correlation between *SOAT1* expression and the clinicopathological characteristics of gliomas was investigated using clinical and transcriptome (RNA-seq) data from TCGA. In addition, correlation analysis was performed between *SOAT1* expression and immune checkpoint genes or chemokines/chemokine receptor genes. Moreover, functional pathways regulated by *SOAT1* were characterized using Gene Ontology (GO) and Kyoto Encyclopedia of Genes and Genomes (KEGG) analysis. To better understand the regulatory mechanism of *SOAT1* in glioma, protein-protein interaction (PPI) network and co-expression analyses of *SOAT1* in glioma were conducted.

## Materials and Methods

### Patient Data and SOAT1 Gene Expression Analysis

Standardized RNA-seq and clinical data of patients with glioma, including 174 patients with GBM and 529 with lower grade glioma [World Health Organization (WHO) grade 2–3; lower grade glioma LGG] were obtained from The Cancer Genome Atlas (TCGA; https://portal.gdc.cancer.gov/). The gene expression level of *SOAT1* in 33 cancer and 31 normal tissues was obtained from the Genotype-Tissue Expression (GTEx) database (https://www.gtexportal.org/home/-index.html). The RNA-seq data was downloaded from UCSC XENA (https://xenabrowser.net/datapages/). The correlation between the *SOAT1* gene expression level and the clinicopathological characteristics of glioma patients in TCGA datasets were investigated. Furthermore, the gene expression levels of *SOAT1* between cancer and normal tissues were analyzed in the integrated datasets (combined TCGA with GTEx databases).

### Survival Analysis

The patients were divided into high- and low-expression groups based on the median value of *SOAT1* expression. Kaplan-Meier curves (K-M curves) and prognostic risk score were used to visualize the significant difference in overall survival (OS) between the high- and low-expression groups. Receiver operating characteristic (ROC) curves were used to show the prognostic value of *SOAT1* in glioma. P-values and hazard ratio (HR) with 95% confidence interval (CI) were generated using log-rank tests and univariate or multivariate Cox regression analyses.

### Correlation Between *SOAT1* Expression and Immune Characteristics

The single-sample GSEA (ssGSEA) algorithm was used to perform Spearman’s correlation analysis between *SOAT1* expression and immune cell infiltration (18) using the R package “GSVA” (19). Cell type-level proportion analysis was also performed using gene expression profiling interactive analysis (GEPIA) 2021 (http://gepia2021.cancer-pku.cn/). Spearman’s correlation analysis was performed to assess the correlation between *SOAT1* expression and immune infiltration through tumor immune estimation resource version 2 (TIMER2.0; http://timer.comp-genomics.org/).

To further elucidate the correlation between immune cell infiltration and *SOAT1* expression in glioma, the TISIDB (an integrated repository portal for tumor-immune system interactions. http://cis.hku.hk/TISIDB/index.php) tool was used to analyze the correlation between *SOAT1* expression and immune cell infiltration. The landscape of correlation between *SOAT1* expression and 28 tumor-infiltrating lymphocytes across multiple cancer types was demonstrated using the “lymphocyte” module. The ESTIMATE algorithm was also used to analyze the immune and stromal scores, to evaluate the tumor immune microenvironment of *SOAT1* expression in glioma. In addition, Pearson’s correlation coefficient was performed to explore the correlation between immune checkpoint gene expression and *SOAT1* expression. To further investigate the association between *SOAT1* expression and immune cell migration, Pearson’s correlation coefficient was performed between *SOAT1* expression and chemokines/chemokine receptor genes based on TCGA cohort.

### Immunofluorescence Assay

The clinicopathological characteristics of 55 patients with gliomas and 6 patients with refractory epilepsy treated by surgery in the Fifth Affiliated Hospital of Zhengzhou University (Zhengzhou Henan China) between January 2016 and December 2020 were retrospectively analyzed, and corresponding paraffin-embedded specimens were collected for immunofluorescence. Among the 55 cases of glioma, 14 cases were WHO grade 2, 21 cases were grade 3, and 20 cases were GBM. The 6 cases of refractory epilepsy were all pathologically diagnosed as gliosis.

Paraffin-embedded specimens were cut into 3-μm sections.

**HE staining**: Paraffin tissue sections were deparaffinized in xylene and rehydrated in degraded ethanol, respectively. After washing with dH_2_O, slides were stained with hematoxylin and eosin solution in sequence followed by being washed with dH_2_O. Then slides were dehydrated in degraded ethanol and immersed in xylene.

**Immunofluorescence assay:** Following deparaffinization with xylene and rehydration, antigen retrieval was performed by microwave treatment in ethylenediaminetetraacetic acid (pH 8.0) antigen retrieval buffer for 23 min. The endogenous peroxidase was blocked with 3% H_2_O_2_. Non-specific binding was blocked for 30 min using bovine serum albumin. The sections were washed in phosphate-buffered saline (PBS; pH 7.4). Diluted primary antibodies against *SOAT1* (sc-69836; Santa Cruz Biotechnology, Inc.) and *FoxP3* (2A11G9l; Santa Cruz Biotechnology, Inc.) were added to the tissue and incubated overnight at 4°C in a humidified chamber. Samples were then incubated with horseradish peroxidase-labelled secondary antibody for 50 min at room temperature in the dark. Sulfo-cyanine 3 tyramide signal amplification (CY3-TSA; G1223, Wuhan Servicebio Technology Co., Ltd.) and fluorescein isothiocyanate (FITC-TSA; G1222, Wuhan Servicebio Technology Co., Ltd.) were used for fluorescence signal amplification and autofluorescence quenchers (G1221; Wuhan Servicebio Technology Co., Ltd.) were used to remove autofluorescence. DAPI (G1012; Wuhan Servicebio Technology Co., Ltd.) was used for nuclear staining. Results were visualized and photographed under a fluorescence microscope (Nikon ECLIPSE C1; Nikon Corporation). Quantitative evaluation was performed by examining each section using at least three different high-power fields to calculate the percentage of positive cells in each section. Positive rate = number of positive cells/total number of cells. The median value of *SOAT1* expression was 4.9%, and the percentage of positive cells in the high-expression group was ≥4.9%, while that in the low-expression group was <4.9%. The statistical summary is presented in **Supplementary Table S3B**. The present study was reviewed and approved by the Ethics Committee of the Fifth Affiliated Hospital of Zhengzhou University. Written informed consent for participation was not required for this study in accordance with the national legislation and the institutional requirements.

### Differentially Expressed Gene (DEG) and Functional Analysis

Patients were divided into the *SOAT1* high- and low-expression groups based on the median *SOAT1* mRNA expression value, and DEG analysis was conducted between these two groups, with adjusted P<0.05 and |log2 (FoldChange)|>2 set as the screening thresholds. To further explore the biological processes (BPs) and signaling pathways involved in *SOAT1*, R package “clusterProfiler” (20) was employed to perform GO and KEGG analyses based on DEG analysis. Adjusted P<0.05 was considered to indicate significant enrichment.

### PPI Network and Co-Expression Analyses

A PPI network was constructed using the Search Tool for the Retrieval of Interacting Genes/Proteins (STRING) database (https://string-db.org/) with the following qualifications: “evidence”, “experiments”, “low confidence (0.150)”, “no more than 50 interactors”. Co-expression analysis was also performed using “R” software with the screening thresholds: |Pearson’s correlation coefficient|>0.6, P-value<0.05. A Venn diagram was used to perform an intersection analysis of the PPI network and co-expression analysis. To further investigate the correlation between *SOAT1* and intersection genes, Pearson’s correlation coefficient was adopted.

### Statistical Analysis

All statistical analysis was performed using R software (version 3.6.3). According to whether the data was normally distributed and whether the variance was uniform, Wilcoxon rank sum test, Welch t-test, or t-test were used to compare the difference in *SOAT1* expression between two different groups, respectively. One-way ANOVA, Welch one-way ANOVA or Kruskal-Wallis test were performed for multi-group comparisons. Kaplan-Meier curves and log-rank tests were performed to compare the difference in survival between the high- and low-expression groups. ROC curves, prognostic risk score, and univariate or multivariate Cox proportional hazards regression analysis were performed to evaluate the prognostic value of *SOAT1* in glioma. P<0.05 was considered to indicate a statistically significant difference.

## Results

### The mRNA Level of *SOAT1* Was Overexpressed in Various Tumors, Including Glioma

**Figure 1:**
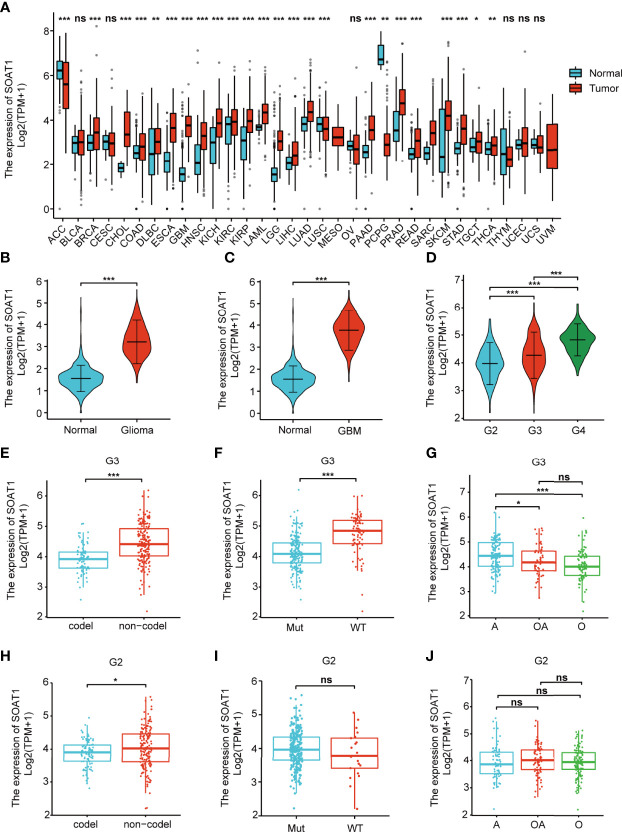
*SOAT1* mRNA was overexpressed in various tumors, including glioma, and the high *SOAT1* expression predicted an advanced malignancy of glioma. **(A)** Pan-cancer analysis was performed to compare the mRNA level of *SOAT1* between tumors and corresponding normal tissues in the integrated datasets (combined TCGA with GTEx databases). **(B)** Expression levels of *SOAT1* in overall glioma and normal tissues from the TCGA and GTEx databases. **(C)** Expression levels of *SOAT1* in GBM and normal tissues from the TCGA and GTEx databases. **(D)** Expression levels of *SOAT1* in different tumor grades from the TCGA dataset. **(E–J)** Expression levels of *SOAT1* in *1p19q* non-co-deleted and *1p19q*-co-deleted types, *IDH* wild and *IDH* mutant types, astrocytoma, and oligodendroglioma and oligoastrocytoma in grade 2/3 gliomas from TCGA dataset. G2, WHO grade 2; G3, WHO grade 3; G4, WHO grade 4; codel, *1p19q*-co-deleted type; non-codel, *1p19q* non-co-deleted type; Mut, *IDH* mutant type; WT, *IDH* wild type. A, astrocytoma; OA, oligoastrocytoma; O, oligodendroglioma; *SOAT1*, *sterol O-acyltransferase 1*; GTEx, genotype-tissue expression; TCGA, The Cancer Genome Atlas; IDH, isocitrate dehydrogenase. ns, P ≥ 0.05; *P < 0.05; **P < 0.01; ***P < 0.001.

A pan-cancer analysis was performed to compare the mRNA level of *SOAT1* between tumor and corresponding normal tissues in the integrated datasets (combined TCGA with GTEx databases), which revealed that *SOAT1* was upregulated in 22/33 tumor types and downregulated in 3 tumor types (**Figure 1A**). In addition, the mRNA level of *SOAT1* was significantly upregulated in both LGG and GBM compared to normal brain tissues (**Figures 1B, C**). Furthermore, *SOAT1* expression was elevated as the tumor grade increased (**Figure 1D**). The *SOAT1* expression of *isocitrate dehydrogenase* (*IDH*) wild type was higher than that of *IDH* mutant type in WHO grade 3 and overall gliomas (**Figure 1F**; **Supplementary Table S1**). *SOAT1* expression in *1p19q* non-co-deleted gliomas was higher than that of *1p19q*-co-deleted gliomas (**Figures 1E**, **H**; **Supplementary Table S1**). Since *IDH* wild type and *1p19q* non-co-deletion are malignant biomarkers of glioma (21), the high *SOAT1* expression may predict the advanced malignancy of glioma. Nevertheless, no difference in *SOAT1* expression was identified between different histological types and *IDH* types in grade 2 gliomas (**Figures 1G, I, J**). In combination, *SOAT1* was widely overexpressed in a variety of tumors, and the high *SOAT1* expression was found to be correlated with malignant clinicopathological characteristics in glioma.

### *SOAT1* Expression Was Associated With Poor Prognosis in All Diffuse Gliomas of All Grades Taken Together

**Figure 2:**
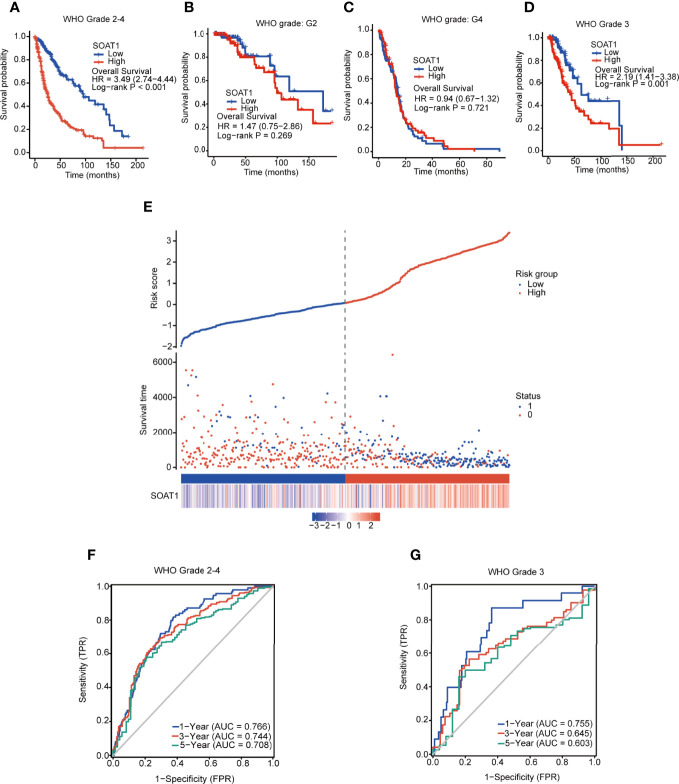
*SOAT1* expression was associated with poor prognosis in all diffuse gliomas of all grades taken together. **(A–D)** KM curves and log-rank tests of OS for *SOAT1* in overall glioma, grade 2 glioma, GBM (grade 4), and grade 3 glioma, based on the TCGA cohort. **(E)** Prognostic risk score of OS for *SOAT1* in overall glioma based on the TCGA cohort. **(F, G)** ROC curve of OS for *SOAT1* in overall and grade 3 glioma, based on the TCGA cohort. *SOAT1*, *sterol O-acyltransferase 1*; KM, Kaplan Meier; OS, overall survival; GBM, glioblastoma; TCGA, The Cancer Genome Atlas; ROC, receiver operating characteristic.

Using K-M curves and log-rank tests, the high *SOAT1* expression was found to be associated with poor prognosis in overall and grade 3 gliomas, but not in grade 2 gliomas and GBM (**Figures 2A–D**). The prognostic risk score demonstrated that the fatality rate in the *SOAT1* high-expression group was significantly higher than that in the low-expression group. The risk score was positively correlated with the *SOAT1* expression level (**Figure 2E**). To further observe the predictive effect of *SOAT1* expression on the OS of glioma patients, ROC curves were employed. It was found that *SOAT1* had a marked predictive ability for the 1-, 3-, and 5-year OS of overall and grade 3 gliomas. The survival area under the curve (AUC) of *SOAT1* expression was 0.766, 0.744, and 0.708 for the 1-, 3-, and 5-year OS, respectively, in overall glioma. The AUC of *SOAT1* expression was 0.755, 0.645, and 0.603 for the 1-, 3-, and 5-year OS, respectively, in WHO grade 3 gliomas (**Figures 2F, G**).

In addition, univariate Cox regression analysis indicated that *SOAT1* was a poor prognostic factor for glioma patients; the HR (95% CI) was 2.863 (2.358-3.477). However, multivariate Cox regression analysis lost statistical significance (**Supplementary Table S2**). Furthermore, according to WHO 2021 classification of brain tumors, we classified gliomas into astrocytoma *IDH*-mutant grade 2, astrocytoma *IDH*-mutant grade 3, oligodendroglioma grade 2, oligodendroglioma grade 3, astrocytoma *IDH* wild type, and GBM in TCGA cohort. We divided patients into high/low groups by the median value of *SOAT1* expression and performed the survival analysis in each type of glioma. Unfortunately, no statistical significance of survival analysis was identified between *SOAT1* -high and -low groups in all types of gliomas (**Supplementary Figure S5**). Only the astrocytoma *IDH* mutant grade 3 showed a little tendency toward poor prognosis in the high-expression group (HR=1.69, 0.82-3.51, P=0.16. **Supplementary Figure 5A**). Therefore, high expression of *SOAT1* was correlated with poor prognosis in glioma, but *SOAT1* was not an independent prognostic factor in glioma.

### *SOAT1* Expression Was Positively Correlated With Immune Infiltration in the TME

**Figure 3:**
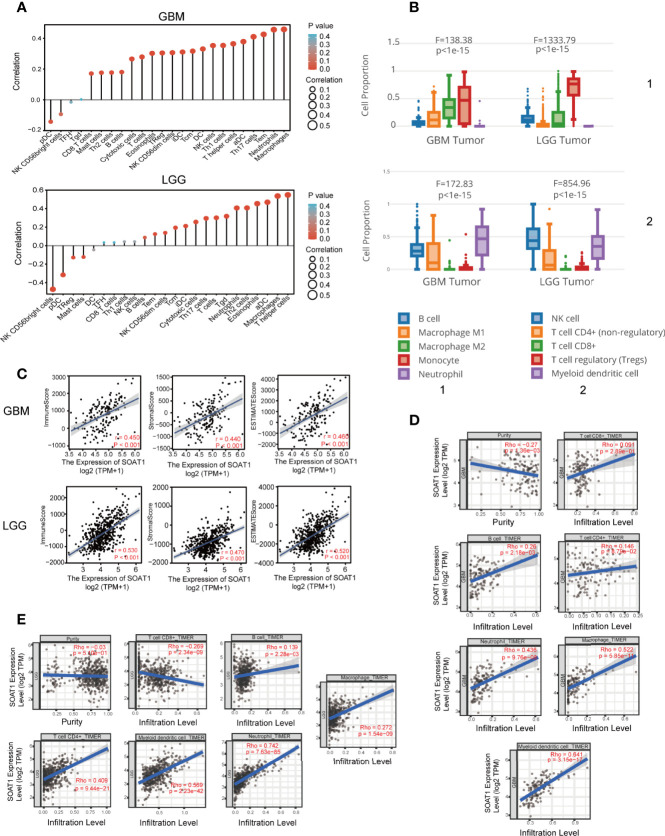
*SOAT1* expression was associated with immune infiltration in the tumor microenvironment. **(A)** Correlation analysis of *SOAT1* expression with immune infiltrating cells in GBM and LGG by ssGSEA algorithm based on the TCGA cohort. **(B)** Relationship between the proportion of immune cell infiltration and the expression level of *SOAT1* from GEPIA2021. **(C)** Correlation analysis of *SOAT1* expression with immune, stromal, and ESTIMATE scores in GBM and LGG. **(D)** Correlation analysis of *SOAT1* expression with purity of tumor, macrophage, neutrophil, myeloid dendritic cell, CD8+ T cell, CD4+ T cell, and B cell in GBM from TIMER2.0. **(E)** Correlation analysis of *SOAT1* expression with purity of tumor, macrophage, neutrophil, myeloid dendritic cell, CD8+ T cell, CD4+ T cell, and B cell in LGG from TIMER2.0. aDC, activated DC; DC, dendritic cells; iDC, immature DC; NK, natural killer; pDC, plasmacytoid DC; Th cells, T helper cells; Tcm, T central memory; Tem, T effector memory; TFH, T follicular helper; Tgd, T gamma delta; Th cells, T helper cells; Treg, T regulatory cells. *SOAT1*, *sterol O-acyltransferase 1*; GBM, glioblastoma; LGG, lower grade glioma; ssGSEA, single-sample GSEA; TCGA, The Cancer Genome Atlas; GEPIA, gene expression profiling interactive analysis.

Immunotherapy, including therapeutic vaccines and engineered T-cells based on tumor-immune cell interactions and blockers, has become an important strategy in the field of cancer research, including glioma (22–26). The relationship between *SOAT1* expression and immune cell infiltration in the TME was determined using the ssGSEA algorithm based on the TCGA cohort. The expression of *SOAT1* was found to be positively correlated with macrophages, neutrophils, T effector memory (Tem), T helper 17 cells (Th17), and activated dendritic cells (aDC), and negatively correlated with plasmacytoid DC (pDC), natural killer (NK), and CD56 bright cells in the GBM (**Figure 3A**). *SOAT1* expression was positively correlated with T helper cells, macrophages, aDC, eosinophils, and T helper 2 cells (Th2), and negatively correlated with NK CD56 bright and pDC cells in the LGG (**Figure 3A**). Next, the relationship between the proportion of immune cell infiltration and the expression level of *SOAT1* was demonstrated by searching the GEPIA2021 database (**Figure 3B**).

To further identify and quantify the immune and matrix components in GBM and LGG, immune, stromal, and ESTIMATE scores were employed, and the results indicated that *SOAT1* expression was positively correlated with all three scores in GBM and LGG (**Figure 3C**). In addition, correlation analysis results for *SOAT1* expression and several tumor infiltrating lymphocytes (TILs) from the TIMER2.0 database are displayed in **Figures 3D, E**. The expression level of *SOAT1* was negatively correlated with purity of tumor in GBM and LGG (r=-0.27, P=1.36e-03, r=-0.03, P=-5.10e-01) (**Figures 3D, E**). Of note, *SOAT1* expression was positively correlated with multiple TILs in GBM, including macrophages (r=0.522, P=5.85e-11), neutrophils (r=0.436, P=9.76e-08), and myeloid dendritic cells (r=0.641, P=3.15e-17). In addition, *SOAT1* expression was positively correlated with multiple TILs in LGG, including CD4+ T cells (r=0.409, P=9.44e-21), myeloid dendritic cells (r=0.569, P=2.23e-42), and neutrophils (r=0.742, P=7.63e-85).

**Figure 4:**
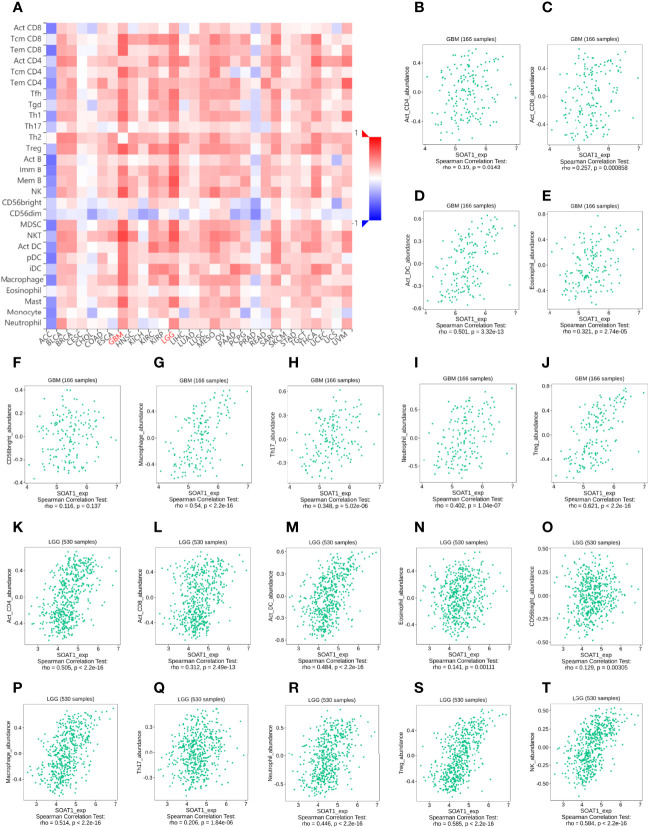
*SOAT1* expression was associated with immune infiltration in the tumor microenvironment. **(A)** Pan-cancer analysis of the correlation between *SOAT1* expression and immune infiltrating cells based on TISIDB dataset. **(B–T)** Correlation between *SOAT1* expression and immune infiltrating cells in GBM and LGG based on TISIDB dataset. aDC, activated DC; DC, dendritic cells; iDC, immature DC; NK, natural killer; pDC, plasmacytoid DC; Th cells, T helper cells; Tcm, T central memory; Tem, T effector memory; TFH, T follicular helper; Tgd, T gamma delta; Th cells, T helper cells; Treg, T regulatory cells; Imm B, immature cell B; Mem B, memory cell B; MDSC, myeloid-derived suppressor cells; *SOAT1*, *sterol O-acyltransferase 1*; LGG, lower grade glioma; TISIDB, an integrated repository portal for tumor-immune system interactions.

To further validate the correlation between *SOAT1* expression and immune infiltration, the correlation between *SOAT1* expression and 28 TILs from the TISIDB database was examined. The pan-cancer analysis of the correlation between *SOAT1* expression and 28 TILs is displayed *via* a heatmap (**Figure 4A**). Specifically, *SOAT1* expression was positively correlated with multiple TILs in GBM and LGG (**Figures 4B–T**; **Supplementary Figures S1** and **S2**). The above results indicated that the expression level of *SOAT1* was positively correlated with various tumor infiltrating immune cells in the TME.

### Correlation Between *SOAT1* Expression and Treg Infiltration Was Not Strong

Treg is a subgroup of CD4+ cells. In previous bioinformatics analysis, *SOAT1* expression was positively correlated with multiple CD4+ T cells and Tregs based on different dataset (**Figures 3A, E**, **Figures 4B, J, K, S**; **Supplementary Figures S1Q, S**, and **Supplementary Figures S2J, R**). *Forkhead Box P3* (*FoxP3*) is a protein-coding gene that is crucial for the development and inhibitory function of Treg (27). *FoxP3* appears to be distinctive for Tregs as it is expressed in CD4+ CD25+ T cells and CD4+ CD25+ CD8- thymocytes whereas it is not found in other thymic cells, T cells, B cells, natural killer cells, or natural killer T cells (28, 29). *Foxp3* also was found to control the expression of gene programs, which define and maintain Treg cell identity and function (30, 31). Therefore, *FoxP3* is reliable and a constant marker that has served to isolate and characterize Tregs. Furthermore, *Foxp3+* Tregs are impacted by different environmental conditions and metabolic differences associated with diverse transcriptional patterns (32). *Foxp3+* Tregs have been extensively studied in gliomas and frequently infiltrate high-grade malignant gliomas (33). Since *FoxP3+* Tregs play a crucial role in glioma-mediated immunosuppression (34), we wonder whether the *SOAT1* expression was associated with immunosuppression in glioma.

**Figure 5:**
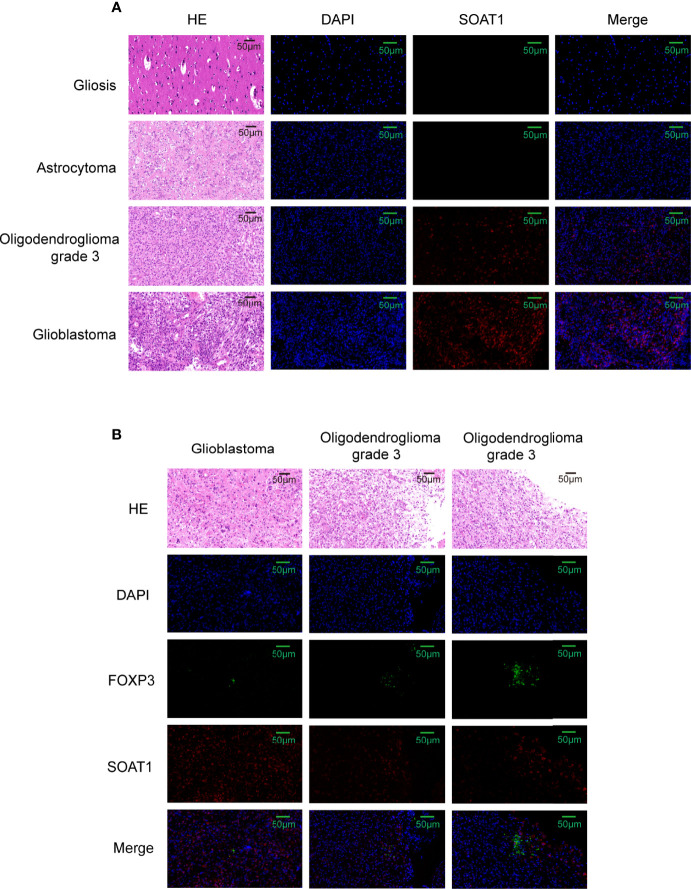
Correlation between *SOAT1* expression and Treg infiltration was not strong. **(A)** Representative HE images and immunofluorescence images of *SOAT1* expression in glioma, astrocytoma grade 2, oligodendroglioma grade 3, glioblastoma. **(B)** The co-localization of high expression of *SOAT1* with *FoxP3+* Treg infiltration in glioma. *SOAT1*, *sterol O-acyltransferase 1*; Treg, T regulatory cell.

To further validate this correlation, immunofluorescence was performed by double-labeling *SOAT1* and *FoxP3* on paraffin-embedded specimens from 55 cases of glioma and 6 of gliosis. Pathologists have made diagnosis based on histology and molecular characteristics. Histologically, among the 55 gliomas, 20 were GBM, 21 were grade 3 gliomas, and 14 were grade 2 gliomas. Of the 21 grade 3 gliomas, 13 were oligodendroglioma grade 3 and 8 were astrocytoma grade 3; among the 14 grade 2 gliomas, 5 were oligodendrogliomas grade 2, and 9 were astrocytoma grade 2. Consistent with our previous study, *SOAT1* expression was increased as the tumor grade increased (**Figure 5A**; **Supplementary Table S3A**), while there was no difference in clinicopathologic features such as age, gender, epilepsy, histological type, preoperative Karnofsky performance status, and tumor diameter between the high- and low-expression groups. The difference of *SOAT1* expression between the astrocytoma grade 2–3 and the oligodendroglioma grade 2–3 did not reach statistical significance (P=0.489) probably due to the small sample size. FoxP3+ Treg infiltration was observed in 17 cases of glioma, mostly in high-grade gliomas (9 GBM, 7 WHO grade 3, and 1 WHO grade 2). Although the difference in *FoxP3+* Treg infiltration between *SOAT1* high- and low-expression groups did not reach statistical significance (χ2 = 0.24, P=0.622), the positive rate of *FoxP3* in the *SOAT1* high-expression group (35.7%) was higher than that in the *SOAT1* low-expression group (25.9%) (**Supplementary Table S3A**). In addition, it was found that some fields of *SOAT1* high-expression group co-localized with *FoxP3+* Treg infiltration (**Figure 5B**). The above results suggest that a positive trend of the correlation between *SOAT1* expression and Treg infiltration was observed in this cohort and some of the Treg cells or tumor cells in their immediate vicinity may be expressing *SOAT1*.

### *SOAT1* Expression Was Associated With Immune Checkpoint Genes and Chemokine/Chemokine Receptor Genes

**Figure 6:**
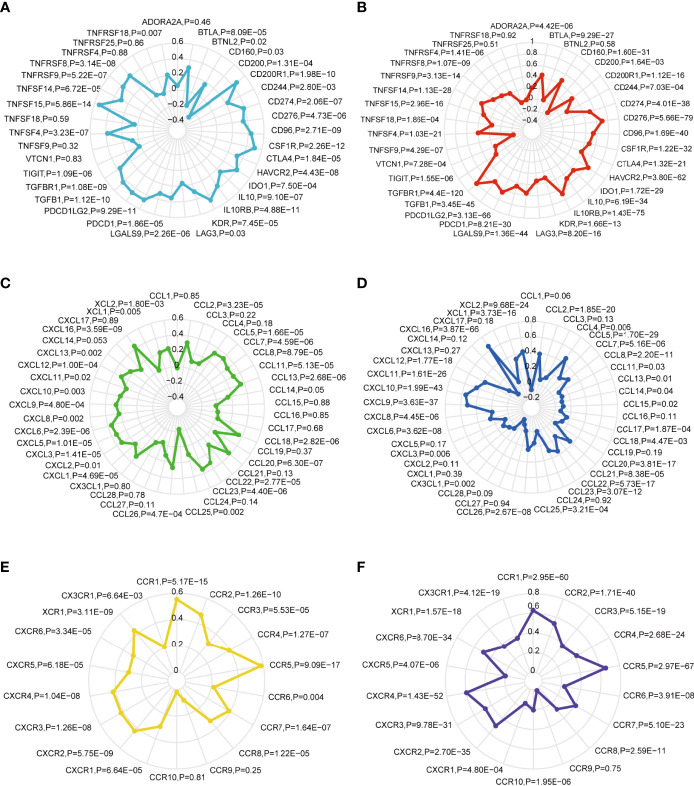
*SOAT1* expression was positively correlated with multiple immune checkpoint genes and chemokine/chemokine receptor genes. **(A)** The Pearson correlation between *SOAT1* expression and immune checkpoint gene levels in GBM based on TCGA cohort. **(B)** Pearson’s correlation coefficient between *SOAT1* expression and immune checkpoint gene levels in LGG based on TCGA cohort. **(C)** Pearson’s correlation coefficient between *SOAT1* expression and chemokine gene levels in GBM based on the TCGA cohort. **(D)** Pearson’s correlation coefficient between *SOAT1* expression and chemokine gene levels in LGG based on the TCGA cohort. **(E)** Pearson’s correlation coefficient between *SOAT1* expression and chemokine receptor gene levels in GBM based on the TCGA cohort. **(F)** Pearson’s correlation coefficient between *SOAT1* expression and chemokine receptor gene levels in LGG based on the TCGA cohort. *SOAT1*, *sterol O-acyltransferase 1*; GBM, glioblastoma; TCGA, The Cancer Genome Atlas; LGG, lower grade glioma.

Immune checkpoints have been proven to be involved in tumor immunosurveillance escape (35). Immunotherapy based on immune checkpoint blockade is increasingly considered as the most promising therapy for GBM in addition to operative treatment (36). Pearson’s correlation coefficient was performed between the *SOAT1* expression and 35 immune checkpoint genes in LGG and GBM based on the TCGA dataset. The results demonstrated that *SOAT1* expression was positively correlated with various checkpoint genes in GBM and LGG (**Figures 6A, B**). In particular, *SOAT1* expression had a significant positive correlation with *PD-L1* (*CD274*, Aliases for *PD-L1* gene) (r=0.52 P=4.01e-38 in LGG, r=0.39 P=2.06e-07 in GBM), monoclonal antibodies against which have been used in clinical trials of GBM (37). These results indicated that *SOAT1* may be involved in tumor immune escape.

Next, the relationship between *SOAT1* expression and multiple chemokine/chemokine receptor gene levels in GBM and LGG was comprehensively analyzed. As shown in **Figures 6C–F**, *SOAT1* expression was positively correlated with multiple chemokine gene levels such as *CCL13* (r=0.35, P=2.68e-06), *CCL20* (r=0.37, P=6.30e-07), and *CCL18* (r=0.35, P=2.82e-06) in GBM, and *CCL5* (r=0.46, P=1.70e-29), *CCL2* (r=0.39, P=1.85e-20), and *CCL20* (r=0.35, P=3.81e-17) in LGG. *SOAT1* expression was also positively correlated with multiple chemokine receptor gene levels, including those of *CCR5* (r=0.58, P=9.09e-17), *CCR1* (r=0.55, P=5.17e-15), and *CCR2* (r=0.47, P=1.26e-10) in GBM, and *CCR5* (r=0.665, P=2.97e-67), *CCR1* (r=0.63P=2.95e-60), and *CCR2* (r=0.53, P=1.71e-40) in LGG. These results indicated that *SOAT1* overexpression may increase the secretion of chemokines to attract immune infiltration into the TME.

### DEG Analysis and Functional Enrichment of *SOAT1* in Glioma

**Figure 7:**
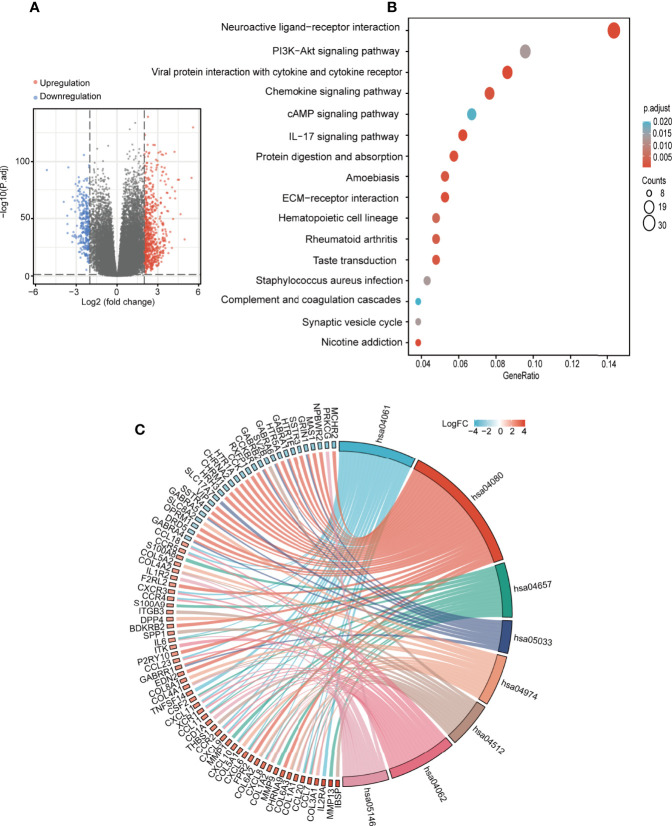
*SOAT1* was involved in a variety of pathways and BPs in glioma. **(A)** Volcano plot of DEGs between the high *SOAT1* and low *SOAT1* expression groups based on TCGA cohort. **(B)** KEGG enrichment analysis of *SOAT1* in glioma based on TCGA cohort. **(C)** Chordal graph of significantly enriched pathways and BPs of *SOAT1* in glioma as shown by KEGG analysis based on TCGA cohort. hsa04080, neuroactive ligand-receptor interaction; hsa04061, viral protein interaction with cytokine and cytokine receptor; hsa04657, IL-17 signaling pathway; hsa05033, nicotine addiction; hsa04974, protein digestion and absorption; hsa04512, ECM-receptor interaction; hsa04062, chemokine signaling pathway; hsa05146, amoebiasis. *SOAT1*, *sterol O-acyltransferase 1*; DEGs, differentially expressed genes; TCGA, The Cancer Genome Atlas; KEGG, Kyoto Encyclopedia of Genes and Genomes; BPs, biological processes.

To further understand the role of *SOAT1* in glioma pathogenesis, DEG analysis was performed based on the TCGA dataset, and the |logFC| and adjusted P-value were visualized using volcano plots (**Figure 7A**). The results showed that 662 genes were upregulated, and 289 genes were downregulated under the screening threshold (adjusted P<0.05 and |log2 (FoldChange)| >2). GO and KEGG analyses based on DEGs analysis demonstrated that a variety of pathways and BPs were significantly enriched, including antigen binding, immunoglobulin complex, humoral immune response (GO analysis; **Supplementary Figure S3A**), neuroactive ligand-receptor interaction, *PI3K/AKT* signaling pathway, viral protein interaction with cytokine and cytokine receptor, chemokine signaling pathway, and *cAMP* signaling pathway (KEGG analysis; **Figure 7B**). To further visualize the genes involved in the significantly enriched pathway, a chordal graph was used (**Figure 7C**; **Supplementary Figures S3B** and **S4A**). These results demonstrated that *SOAT1* was involved in a variety of BPs and signaling pathways in the pathogenesis of glioma.

### PPI Network and Co-Expression Analysis of *SOAT1* in Glioma

**Figure 8:**
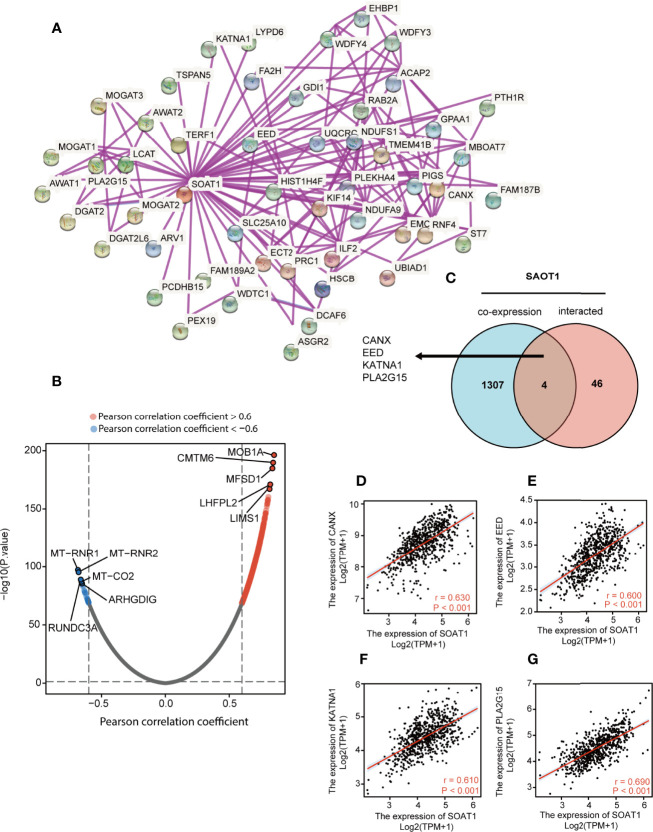
PPI network and co-expression analysis of *SOAT1* in glioma. **(A)** A PPI network of *SOAT1* was constructed using the STRING website. **(B)** Co-expression analysis of *SOAT1* in glioma based on the TCGA cohort **(C)** An intersection analysis of *SOAT1* co-expression analysis and PPI network was performed by Venn diagram. **(D–G)** Pearson’s correlation coefficient of *CANX, EED, KATNA1*, and *PLA2G15* with *SOAT1* expression in glioma, respectively. PPI, protein-protein interaction; *CANX*, calnexin; *EED*, embryonic ectoderm development; *KATNA1*, katanin catalytic subunit A1; *PLA2G15*, phospholipase A2 group XV; STRING, Search Tool for the Retrieval of Interacting Genes/Proteins; TCGA, The Cancer Genome Atlas; *SOAT1*, *sterol O-acyltransferase 1*.

A PPI network was constructed using the STRING website and the 50 identified *SOAT1*-binding proteins (**Figure 8A**). Subsequently, a co-expression analysis of *SOAT1* in glioma was performed using Pearson’s correlation coefficient, and the results showed that 1287 genes were positively correlated, and 24 genes were negatively correlated with *SOAT1* expression in glioma under the screening threshold (adjusted P<0.05 and |Pearson’s correlation coefficient|>0.6). In addition, the top 5 positively correlated genes were MOB kinase activator 1A (*MOB1A*), CKLF like MARVEL transmembrane domain containing 6 (*CMTM6*), major facilitator superfamily domain containing 1 (*MFSD1*), *LHFPL* tetraspan subfamily member *2 (LHFPL2)*, and *LIM* and senescent cell antigen-like-containing (*LIMS1*), and the top 5 negatively correlated genes were mitochondrially encoded 12S RRNA (*MT-RNR1*), mitochondrially encoded 16S RRNA (*MT-RNR2*), mitochondrially encoded cytochrome C oxidase II (*MT-CO2*), Rho GDP dissociation inhibitor gamma (*ARHGDIG*), and RUN domain containing 3A (*RUNDC3A*) (**Figure 8B**).

The Venn diagram showed that 4 genes were shared between the PPI network and the co-expression analysis: Calnexin (*CANX*), embryonic ectoderm development (*EED*), katanin catalytic subunit A1 (*KATNA1*), and phospholipase A2 group XV (*PLA2G15*) (**Figure 8C**). The correlations between *SOAT1* expression in glioma were demonstrated in **Figures 8D–G**. *CANX* is a calcium-binding, endoplasmic reticulum-associated protein that interacts transiently with newly synthesized N-linked glycoproteins, facilitating protein folding and assembly. The interaction between *SOAT1* and *CANX* was detected by co-sedimentation *via* a density gradient assay (38). Embryonic ectoderm development encodes a member of the polycomb-group (*PcG*) family. *PcG* family members form multimeric protein complexes, which are involved in maintaining the transcriptional repressive state of genes over successive cell generations, and the interaction between *SOAT1* and *CANX* was detected by affinity chromatography technology assay (39). *KATNA1* is the catalytic subunit of a complex which severs microtubules in an ATP-dependent manner. The interaction between *SOAT1* and *KATNA1* was detected using an anti-tag coimmunoprecipitation assay (https://www.ebi.ac.uk/). *PLA2G15* encodes the protein that hydrolyzes lysophosphatidylcholine to glycerophosphorylcholine and a free fatty acid. The interaction between *SOAT1* and *PLA2G15* was detected by genetic interference assay (40). In combination, these genes may play a significant role in the regulatory mechanism of *SOAT1* in gliomas.

## Discussion

*SOAT1* was found to be overexpressed in glioma, compared to normal brain tissue, which was consistent with the report by Geng et al. In addition, it was found that the high *SOAT1* expression was associated with malignant pathological characteristics in glioma, indicating that lipid metabolism was linked to glioma heterogeneity and excessive lipid metabolism indicated a malignant subtype. Geng et al. also found that LDs were correlated with poor survival in patients with GBM. Therefore, the prognostic value of *SOAT1* in glioma was comprehensively explored. Not surprisingly, *SOAT1* expression was associated with poor prognosis in all gliomas taken together but it has no prognostic significance in individual glioma type and grade. Our evidence showed the *SOAT1* higher expression in higher grade and astrocytoma phenotype may explain the association of *SOAT1* with poor prognosis in glioma.

Numerous studies have shown that decreased Treg infiltration reflects an attenuated immunosuppression microenvironment and was correlated with favorable prognosis (34, 41–43). However, the clinical prognostic value of *FoxP3+* Tregs in glioma remains controversial. Studies have shown that Treg infiltration predicted a poor prognosis in glioma (44, 45), while others found there was no correlation between Tregs and glioma prognosis (33, 46). In addition, *FoxP3+* Tregs are most frequently found in GBM and very rarely found in low-grade astrocytomas and oligodendroglial tumors (33, 47). The present study came to similar conclusions; however, some *FoxP3+* Treg infiltration was still observed in grade 3 gliomas and oligodendroglial tumors. Furthermore, we also found that correlation between *SOAT1* expression and Treg infiltration was not strong in glioma. Since it is a small cohort of 55 cases with different tumors of different grades, definite conclusions cannot be drawn based on the limited data. A larger cohort may be required to conclusively prove *FoxP3* association with *SOAT1* expression.

In addition to Tregs, multiple immune cell infiltration and chemokines/chemokine receptors were found to be positively correlated with *SOAT1* expression in glioma, such as macrophages, *CCL16*, and *CCL20*. In fact, increasing evidence shows that there is a strong connection between the tumor metabolic microenvironment and the immune microenvironment. *Farnesyl diphosphate synthase* (*FDPS*) is a key enzyme in isoprenoid biosynthesis. Chen et al. found that *FDPS* promoted glioma proliferation and macrophage recruitment by regulating *CCL20 via* the Wnt/β-catenin signaling pathway (48). The unsaturated fatty acid 20-hydroxyeicosatetraenoic acid, a catalytic product of cytochrome *P450 4A* (*CYP4A*), along with *VEGF* and *TGF-β*, promoted angiogenesis by enhancing the migration of glioma-associated macrophages (GAMs), and inhibition of *CYP4A* prolonged survival and normalized tumor vasculature in glioma (49). A recent study found that extracellular lipid-loading promoted GAM infiltration and neovascularization in tumors, an effect that was augmented by an elevated, continuous supply of systemic lipids (50). This is direct evidence that LD^+^ GBM cells are associated with the infiltration of immune-suppressive phenotype GAMs. Since LD is formed by the aggregation of CEs, it is not surprising that the expression of *SOAT1* is associated with M2 macrophage infiltration in glioma. The above studies demonstrated that lipid metabolic reprogramming contributes toward an immune-suppressive phenotype in glioma.

Studies have found that *SOAT1* acts an oncogene through multiple pathways. The downregulation of *SOAT1* has been found to suppress the proliferation and migration of hepatocellular carcinoma cells by reducing the cholesterol content of the plasma membrane, and then inhibiting the integrin and *TGF-β* signaling pathways (51). Similarly, integrin binding was also significantly enriched (adjusted P=0.021), as shown by the GO and KEGG analyses of upregulated DEGs in glioma (**Supplementary Table S3**). *SOAT1* inhibition can inhibit pancreatic ductal adenocarcinoma (PDAC) progression by activating cholesterol-mediated negative feedback of the mevalonate pathway (13). Xu et al. demonstrated that *SOAT1* inhibition upregulated *Wnt/PCP-YAP* signaling by elevating cellular cholesterol in colon cancer. Nystatin, an inhibitor of cholesterol, synergizes with targeting *SOAT1* in suppressing the viability of colon cancer cells (52). In addition, CE was found to activate the *AKT/mTOR* pathway and promotes tumor growth in breast cancer (53).

*CMTM6* is a membrane protein that co-localizes with the immune checkpoint molecule *PD-L1* on the cell membrane to protect *PD-L1* from degradation, thereby promoting tumor immune escape. Domschke et al. found that silencing *CMTM6* reduced macrophage LDL-uptake (54), suggesting that *CMTM6* was involved in the regulation of cholesterol metabolism. Consistently, our analysis showed that *CMTM6* expression was strongly correlated with *SOAT1* expression in glioma, further evidence revealing the connection between metabolic remodeling and tumor immune escape. *LIMS1* is an adaptor protein that contains five *LIM* domains and is likely involved in integrin signaling. Huang et al. demonstrated that *LIMS1* promoted pancreatic cancer cell survival under oxygen-glucose deprivation conditions by activating *AKT/mTOR* signaling and enhancing *HIF1A* protein translation (55). The present study showed that *LIMS1* was strongly correlated with *SOAT1* expression and may also exert the same function by regulating lipid metabolism. In addition, *SOAT1* was found to be expressed under diverse regulatory mechanisms in tumors. *Runt*-related transcription factor 1 promoted the expression of *SOAT1* by binding to the promoter region of *SOAT1* in squamous cell carcinomas (56). The *p53* loss of heterozygosity can promote *SOAT1* expression by enhancing the transcription of *SOAT1* in PDAC (13). Furthermore, *β-catenin* can directly bind to *SOAT1* promoter element, promoting *SOAT1* transcription in colorectal cancer (57).

In the present study, the prognostic value of *SOAT1* in glioma and its association with immune cell infiltration was elucidated. In addition, various BPs and signaling pathways that *SOAT1* may be involved in during glioma pathogenesis were explored. Finally, multiple genes that may interact with *SOAT1* in gliomas were identified *via* PPI network and co-expression analyses. Therefore, *SOAT1* may serve as a novel target that drives the development of novel strategies of immunotherapy and metabolic therapy.

## Data Availability Statement

The datasets presented in this study can be found in online repositories. The names of the repository/repositories and accession number(s) can be found in the article/**Supplementary Material**.

## Ethics Statement

The studies involving human participants were reviewed and approved by Ethics Committee of the Fifth Affiliated Hospital of Zhengzhou University. Written informed consent for participation was not required for this study in accordance with the national legislation and the institutional requirements.

## Author Contributions

XG participated in the research design and experimental implementation, statistical analysis, and article writing. SZ and ZY provided guidance, funding support, and help to obtain materials for the study. WH, LD, and Z-AL participated in the collection of clinical data. WL provided guidance for the study. XW provided guidance on research design, as well as technical, material, and financial support for the study. All authors agree to be accountable for the content of the work. All authors read and approved the final manuscript.

## Funding

This study was supported by the National Natural Science Foundation of China: 81972361, 81874068. and the Henan Province Science and Technology Research Project: 222102310039.

## Conflict of Interest

The authors declare that the research was conducted in the absence of any commercial or financial relationships that could be construed as a potential conflict of interest.

## Publisher’s Note

All claims expressed in this article are solely those of the authors and do not necessarily represent those of their affiliated organizations, or those of the publisher, the editors and the reviewers. Any product that may be evaluated in this article, or claim that may be made by its manufacturer, is not guaranteed or endorsed by the publisher.

## Supplementary Material

Supplementary Figure 1
**(A–S)** Correlation between *SOAT1* expression and immune infiltrating cells in GBM based on TISIDB dataset. *SOAT1, sterol O-acyltransferase 1*; GBM, glioblastoma; TISIDB, an integrated repository portal for tumor-immune system interactions.

Supplementary Figure 2
**(A–R)** Correlation between *SOAT1* expression and immune infiltrating cells in LGG based on TISIDB dataset. *SOAT1, sterol O-acyltransferase 1*; LGG, lower grade glioma; TISIDB, an integrated repository portal for tumor-immune system interactions.

Supplementary Figure 3
**(A)** GO enrichment analysis of *SOAT1* in glioma based on the TCGA cohort. **(B)** Chordal graph of significantly enriched BP of *SOAT1* in glioma by GO analysis based on TCGA cohort. GO:0006958 complement activation, classical pathway. GO:0002455 humoral immune response mediated by circulating immunoglobulin. GO:0006956 complement activation. GO:0006959 humoral immune response. GO, Gene Ontology; *SOAT1, sterol O-acyltransferase 1*; TCGA, The Cancer Genome Atlas; BP, biological processes.

Supplementary Figure 4
**(A)** Chordal graph of significantly enriched MF and CC of *SOAT1* in glioma by GO analysis based on TCGA cohort. GO:0019814 immunoglobulin complex, GO:0042571 immunoglobulin complex circulating, GO:0003823 antigen binding, GO:0034987 immunoglobulin receptor binding. MF, molecular functions; CC, cellular component; *SOAT1, sterol O-acyltransferase 1*; GO, Gene Ontology; TCGA, The Cancer Genome Atlas.

Supplementary Figure 5
**(A–F)** K-M curves of each type of glioma based on WHO 2021 classification of brain tumors in the TCGA cohort. K-M, Kaplan-Meier; WHO, World Health Organization; TCGA, The Cancer Genome Atlas.
