# Supplementary figures and images for: Comprehensive Analysis of Sterol O-Acyltransferase 1 as a Prognostic Biomarker and Its Association With Immune Infiltration in Glioma
Source: Front Oncol. 2022 May 12;12:896433. doi: 10.3389/fonc.2022.896433 (PMC9133349; doi:10.3389/fonc.2022.896433)

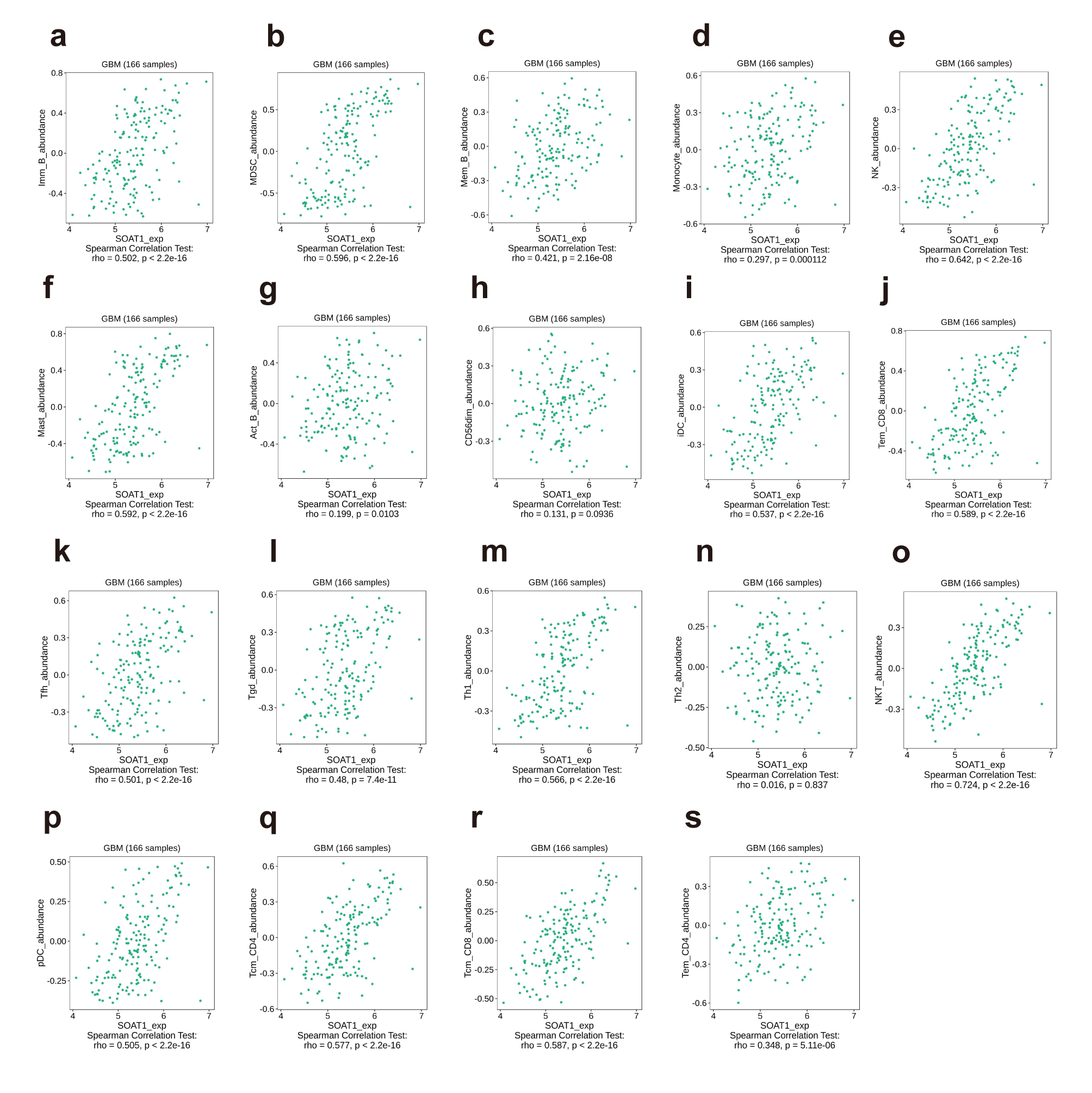

Supplement: Supplementary Figure 1 — (A–S) Correlation between SOAT1 expression and immune infiltrating cells in GBM based on TISIDB dataset. SOAT1, sterol O-acyltransferase 1; GBM, glioblastoma; TISIDB, an integrated repository portal for tumor-immune system interactions. [file Image_1.tif]

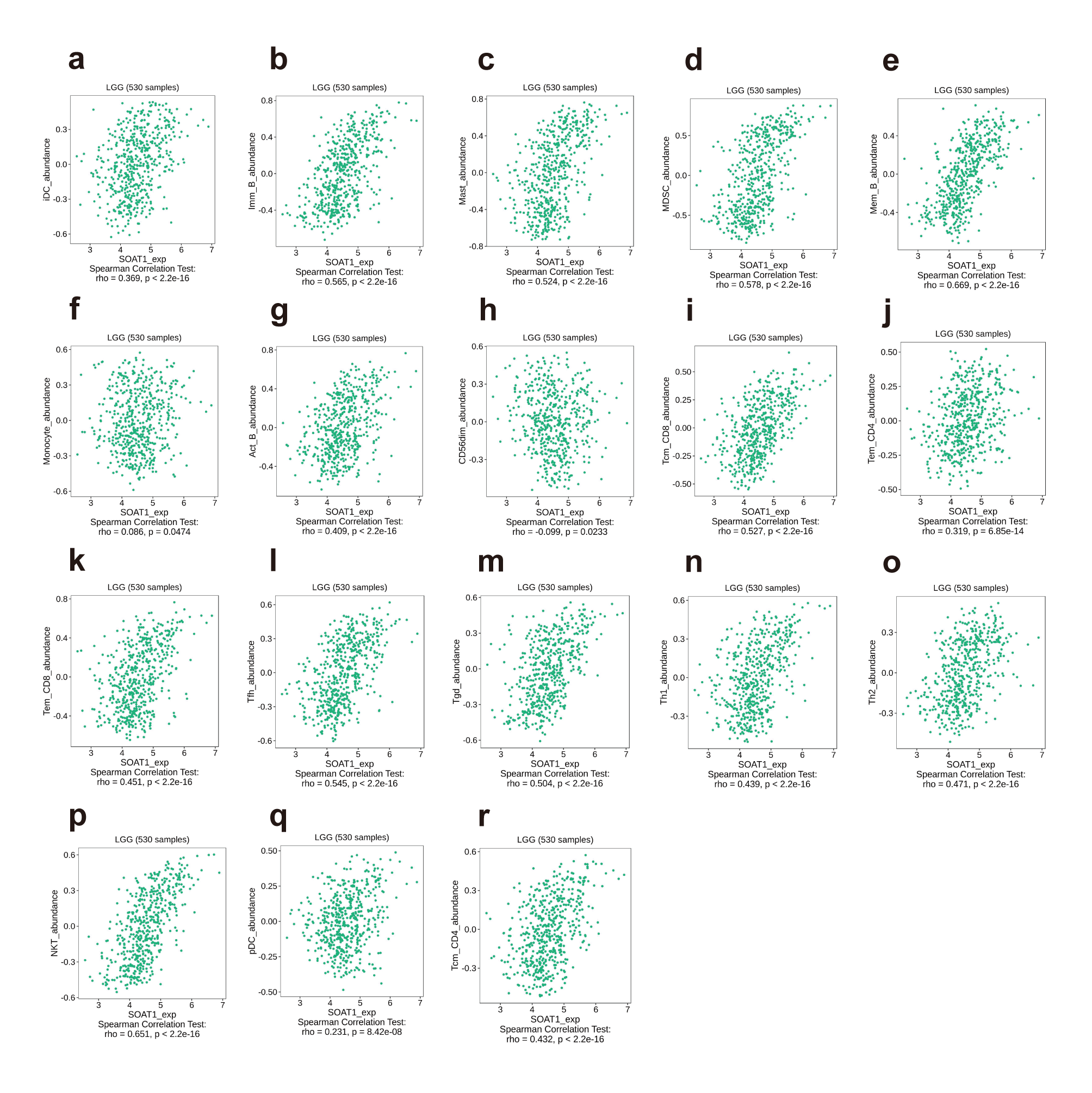

Supplement: Supplementary Figure 2 — (A–R) Correlation between SOAT1 expression and immune infiltrating cells in LGG based on TISIDB dataset. SOAT1, sterol O-acyltransferase 1; LGG, lower grade glioma; TISIDB, an integrated repository portal for tumor-immune system interactions. [file Image_2.tif]

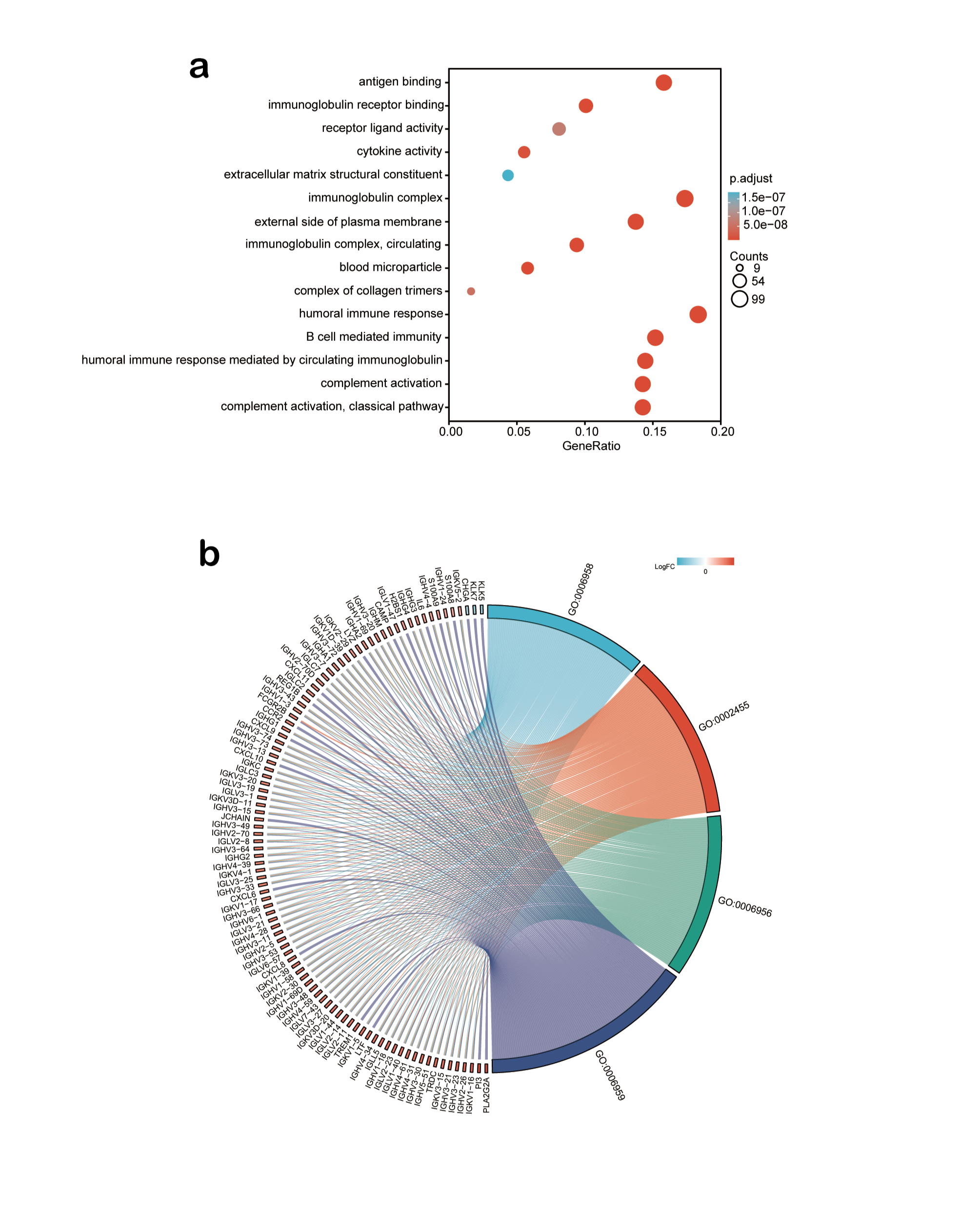

Supplement: Supplementary Figure 3 — (A) GO enrichment analysis of SOAT1 in glioma based on the TCGA cohort. (B) Chordal graph of significantly enriched BP of SOAT1 in glioma by GO analysis based on TCGA cohort. GO:0006958 complement activation, classical pathway. GO:0002455 humoral immune response mediated by circulating immunoglobulin. GO:0006956 complement activation. GO:0006959 humoral immune response. GO, Gene Ontology; SOAT1, sterol O-acyltransferase 1; TCGA, The Cancer Genome Atlas; BP, biological processes. [file Image_3.tif]

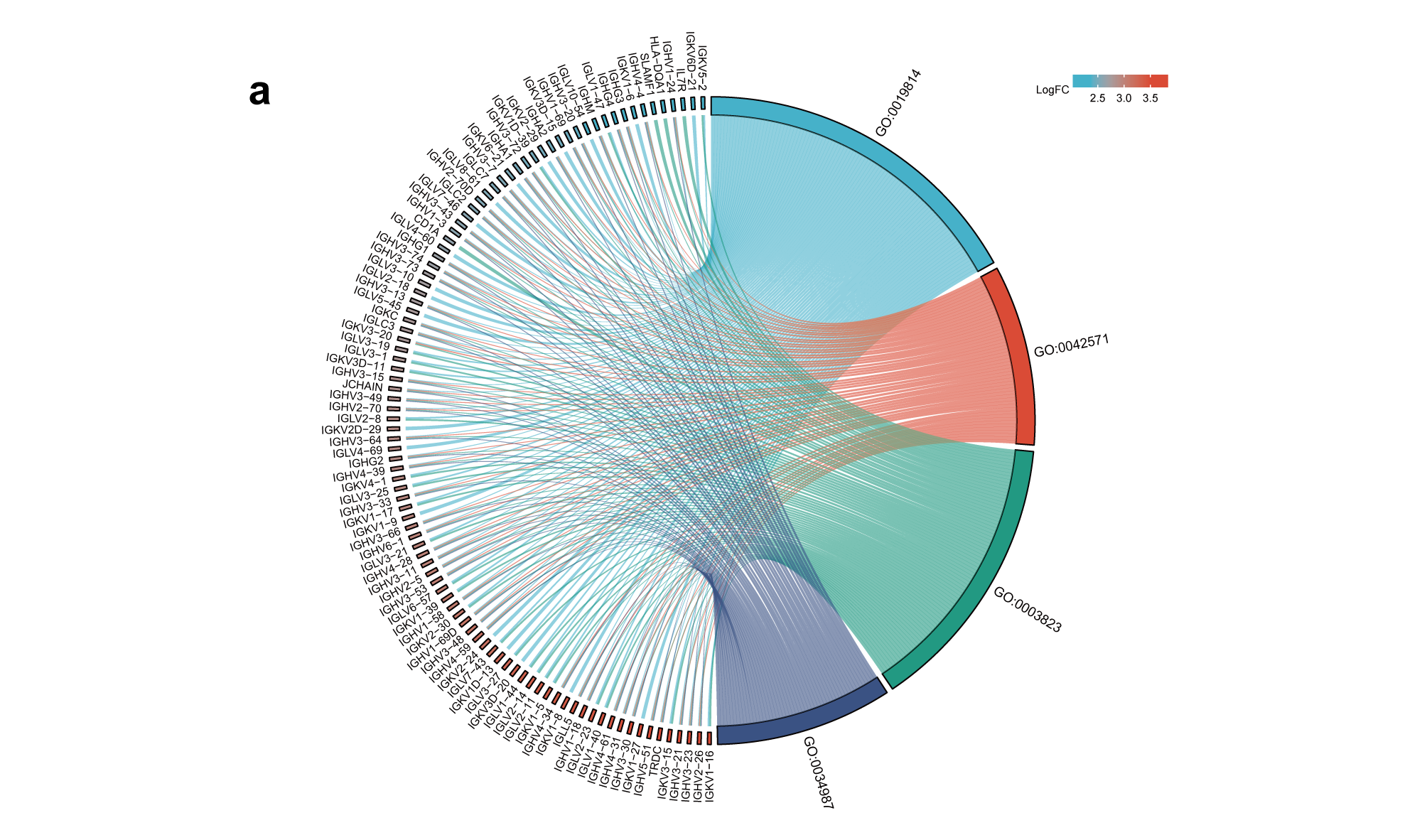

Supplement: Supplementary Figure 4 — (A) Chordal graph of significantly enriched MF and CC of SOAT1 in glioma by GO analysis based on TCGA cohort. GO:0019814 immunoglobulin complex, GO:0042571 immunoglobulin complex circulating, GO:0003823 antigen binding, GO:0034987 immunoglobulin receptor binding. MF, molecular functions; CC, cellular component; SOAT1, sterol O-acyltransferase 1; GO, Gene Ontology; TCGA, The Cancer Genome Atlas. [file Image_4.tif]

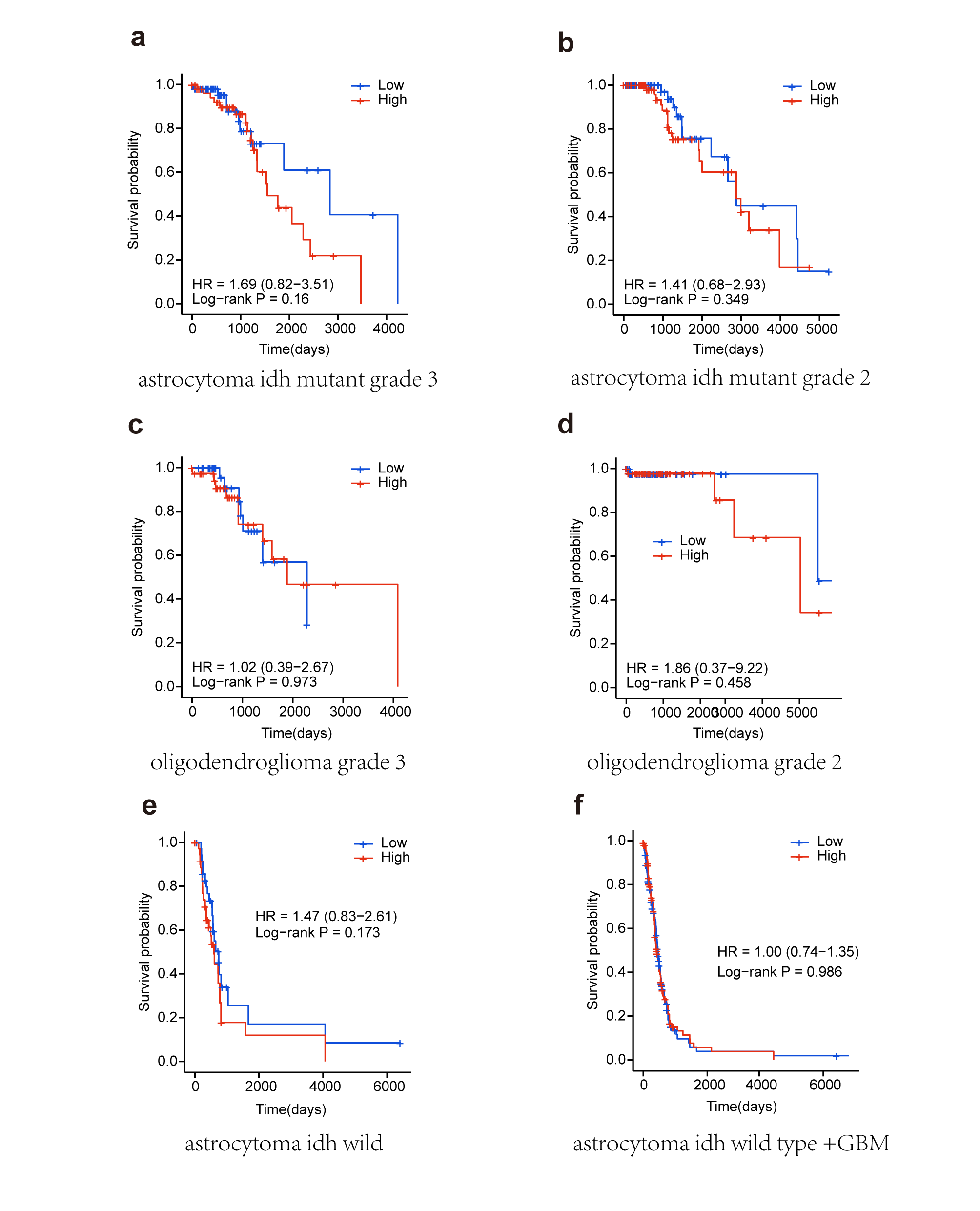

Supplement: Supplementary Figure 5 — (A–F) K-M curves of each type of glioma based on WHO 2021 classification of brain tumors in the TCGA cohort. K-M, Kaplan-Meier; WHO, World Health Organization; TCGA, The Cancer Genome Atlas. [file Image_5.tif]
