# Supplementary material for: Comprehensive Analysis of Sterol O-Acyltransferase 1 as a Prognostic Biomarker and Its Association With Immune Infiltration in Glioma
Source: Front Oncol. 2022 May 12;12:896433. doi: 10.3389/fonc.2022.896433 (PMC9133349; doi:10.3389/fonc.2022.896433)
Supplement: Supplementary file 8 [file Table_1.docx]

| **Characteristic** | **Low expression of SOAT1** | **High** **expression of SOAT1** | **p** | **χ2** | **method** |
| --- | --- | --- | --- | --- | --- |
| n | 348 | 348 |  |  |  |
| WHO grade, n (%) |  |  | < 0.001 | 124.53 | Chisq.test |
| G2 | 159 (25%) | 65 (10.2%) |  |  |  |
| G3 | 123 (19.4%) | 120 (18.9%) |  |  |  |
| G4 | 24 (3.8%) | 144 (22.7%) |  |  |  |
| IDH status, n (%) |  |  | < 0.001 | 149.76 | Chisq.test |
| WT | 46 (6.7%) | 200 (29.2%) |  |  |  |
| Mut | 298 (43.4%) | 142 (20.7%) |  |  |  |
| 1p/19q codeletion, n (%) |  |  | < 0.001 | 103.33 | Chisq.test |
| codel | 144 (20.9%) | 27 (3.9%) |  |  |  |
| non-codel | 202 (29.3%) | 316 (45.9%) |  |  |  |
| Primary therapy outcome, n (%) |  |  | < 0.001 | 29.86 | Chisq.test |
| PD | 45 (9.7%) | 67 (14.5%) |  |  |  |
| SD | 91 (19.7%) | 56 (12.1%) |  |  |  |
| PR | 42 (9.1%) | 22 (4.8%) |  |  |  |
| CR | 102 (22.1%) | 37 (8%) |  |  |  |
| Gender, n (%) |  |  | 0.939 | 0.01 | Chisq.test |
| Female | 150 (21.6%) | 148 (21.3%) |  |  |  |
| Male | 198 (28.4%) | 200 (28.7%) |  |  |  |
| Race, n (%) |  |  | 0.273 | 2.6 | Chisq.test |
| Asian | 6 (0.9%) | 7 (1%) |  |  |  |
| Black or African American | 12 (1.8%) | 21 (3.1%) |  |  |  |
| White | 322 (47.1%) | 315 (46.1%) |  |  |  |
| Age, n (%) |  |  | < 0.001 | 23.8 | Chisq.test |
| <=60 | 303 (43.5%) | 250 (35.9%) |  |  |  |
| >60 | 45 (6.5%) | 98 (14.1%) |  |  |  |
| Histological type, n (%) |  |  | < 0.001 | 134.65 | Chisq.test |
| Astrocytoma | 95 (13.6%) | 100 (14.4%) |  |  |  |
| Glioblastoma | 24 (3.4%) | 144 (20.7%) |  |  |  |
| Oligoastrocytoma | 86 (12.4%) | 48 (6.9%) |  |  |  |
| Oligodendroglioma | 143 (20.5%) | 56 (8%) |  |  |  |
| OS event, n (%) |  |  | < 0.001 | 85.46 | Chisq.test |
| Alive | 272 (39.1%) | 152 (21.8%) |  |  |  |
| Dead | 76 (10.9%) | 196 (28.2%) |  |  |  |
| DSS event, n (%) |  |  | < 0.001 | 81.16 | Chisq.test |
| Alive | 277 (41%) | 154 (22.8%) |  |  |  |
| Dead | 68 (10.1%) | 176 (26.1%) |  |  |  |
| PFI event, n (%) |  |  | < 0.001 | 58.63 | Chisq.test |
| Alive | 226 (32.5%) | 124 (17.8%) |  |  |  |
| Dead | 122 (17.5%) | 224 (32.2%) |  |  |  |
| Age, meidan (IQR) | 42 (33, 53) | 51 (36, 62) | < 0.001 | 46548 | Wilcoxon |
