# Supplementary material for: Comprehensive Analysis of Sterol O-Acyltransferase 1 as a Prognostic Biomarker and Its Association With Immune Infiltration in Glioma
Source: Front Oncol. 2022 May 12;12:896433. doi: 10.3389/fonc.2022.896433 (PMC9133349; doi:10.3389/fonc.2022.896433)
Supplement: Supplementary file 9 [file Table_2.docx]

| **Characteristics** | **Total(N)** | **Univariate analysis** | |  | **Multivariate analysis** | |
| --- | --- | --- | --- | --- | --- | --- |
|  |  | **Hazard ratio (95% CI)** | **P value** |  | **Hazard ratio (95% CI)** | **P value** |
| WHO grade | 634 |  |  |  |  |  |
| G2 | 223 | Reference |  |  |  |  |
| G3 | 243 | 2.999 (2.007-4.480) | **<0.001** |  | 1.928 (1.257-2.956) | **0.003** |
| G4 | 168 | 18.615 (12.460-27.812) | **<0.001** |  | 3.520 (2.068-5.991) | **<0.001** |
| 1p/19q codeletion | 688 |  |  |  |  |  |
| codel | 170 | Reference |  |  |  |  |
| non-codel | 518 | 4.428 (2.885-6.799) | **<0.001** |  | 1.836 (1.095-3.077) | **0.021** |
| Age | 695 | 1.066 (1.056-1.076) | **<0.001** |  | 1.035 (1.023-1.047) | **<0.001** |
| Gender | 695 |  |  |  |  |  |
| Female | 297 | Reference |  |  |  |  |
| Male | 398 | 1.262 (0.988-1.610) | 0.062 |  | 1.153 (0.881-1.509) | 0.300 |
| IDH status | 685 |  |  |  |  |  |
| WT | 246 | Reference |  |  |  |  |
| Mut | 439 | 0.117 (0.090-0.152) | **<0.001** |  | 0.399 (0.258-0.616) | **<0.001** |
| SOAT1 | 695 | 2.863 (2.358-3.477) | **<0.001** |  | 1.247 (0.959-1.622) | 0.100 |
