# Supplementary material for: Comprehensive Analysis of Sterol O-Acyltransferase 1 as a Prognostic Biomarker and Its Association With Immune Infiltration in Glioma
Source: Front Oncol. 2022 May 12;12:896433. doi: 10.3389/fonc.2022.896433 (PMC9133349; doi:10.3389/fonc.2022.896433)
Supplement: Supplementary file 10 [file Table_3.docx]

| **Characteristic** | **High expression of SOAT1** | **low** **expression of SOAT1** | **p** | **χ2** | **method** |
| --- | --- | --- | --- | --- | --- |
| n(%) | 28(50.9%) | 27(49.1%) |  |  |  |
| Gender, n (%) |  |  | 0.916 | 0.01 | Chisq.test |
| Female | 10 (35.7%) | 11 (40.7%) |  |  |  |
| male | 18 (64.3%) | 16 (59.3%) |  |  |  |
| age, n (%) |  |  | 1.000 | 0 | Chisq.test |
| <=60 | 20 (71.4%) | 20 (74.1%) |  |  |  |
| >60 | 8 (28.6%) | 7 (25.9%) |  |  |  |
| WHO grade, n (%) |  |  | **0.026** | **7.32** | Chisq.test |
| 2 | 5 (17.9%) | 9 (33.3%) |  |  |  |
| 3 | 8 (28.6%) | 13 (48.1%) |  |  |  |
| GBM | 15 (53.6%) | 5 (18.5%) |  |  |  |
| histological type, n (%) |  |  | 0.489 | - | Fisher.test |
| Astrocytoma grade 2-3 | 5 (14.3%) | 12 (34.3%) |  |  |  |
| Oligodendroglioma grade 2-3 | 8 (22.9%) | 10 (28.6%) |  |  |  |
| epilepsy, n (%) |  |  | 0.928 | 0.01 | Chisq.test |
| Negative | 20 (71.4%) | 18 (66.7%) |  |  |  |
| positive1 | 8 (28.6%) | 9 (33.3%) |  |  |  |
| Preoperative KPS, n (%) |  |  | 0.395 | 0.72 | Chisq.test |
| >=70 | 19 (67.9%) | 22 (81.5%) |  |  |  |
| <70 | 9 (32.1%) | 5 (18.5%) |  |  |  |
| diameter, n (%) |  |  | 1.000 | 0 | Chisq.test |
| <=5 | 20 (71.4%) | 20 (74.1%) |  |  |  |
| >5 | 8 (28.6%) | 7 (25.9%) |  |  |  |
| foxp3, n (%) |  |  | 0.622 | 0.24 | Chisq.test |
| negetive | 18 (64.3%) | 20 (74.1%) |  |  |  |
| positive | 10 (35.7%) | 7 (25.9%) |  |  |  |

Table 3a. Correlation between SOAT1 expression and pathological features in 55 gliomas

Table 3b. Statistical summary for the SOAT1 staining results

| WHO Grade | case number | SOAT1 expression (%) | | | |
| --- | --- | --- | --- | --- | --- |
|  |  | maximum | minimum | median | mean |
| Glioma | 55 | 88.6 | 0 | 4.9 | 15.52 |
| 2 | 14 | 72.3 | 0 | 1 | 9.19 |
| 3 | 21 | 88.6 | 0 | 2.3 | 14.45 |
| 4 | 20 | 85 | 0 | 9.7 | 21.08 |
| Gliosis | 6 | 70 | 0 | 0 | 20 |
